# Multi-Objective Optimization of a Mine Water Reuse System Based on Improved Particle Swarm Optimization

**DOI:** 10.3390/s21124114

**Published:** 2021-06-15

**Authors:** Yang Liu, Zihang Zhang, Lei Bo, Dongxu Zhu

**Affiliations:** School of Mechanical Electronic & Information Engineering, China University of Mining and Technology (Beijing), Beijing 100083, China; liuyangebox@126.com (Y.L.); zhangzihang414@163.com (Z.Z.); 18811786056@163.com (D.Z.)

**Keywords:** mine water dispatching, improved particle swarm optimization algorithm, mine water reuse

## Abstract

This paper proposes a general hierarchical dispatching strategy of mine water, with the aim of addressing the problems of low reuse rate of coal mine water, and insufficient data analysis. First of all, water quality and quantity data of the Narim River No. 2 mine were used as the research object; the maximum reuse rate of mine water and the system operation rate comprised the objective function; and mine water quality information, mine water standard, and mine water treatment speed were the constraints. A multi-objective optimization scheduling mathematical model of water supply system was established. Then, to address the problems of premature convergence and ease of falling into a local optimum in the iterative process of particle swarm optimization, the basic particle swarm optimization was improved. Using detailed simulation research, the superiority of the improved algorithm was verified. Eventually, the mine water grading dispatching strategy proposed in this paper is compared with the traditional dispatching method. The results show that the hierarchical dispatching system can significantly improve the mine water reuse rate and system operating efficiency.

## 1. Introduction

**Figure 1:**
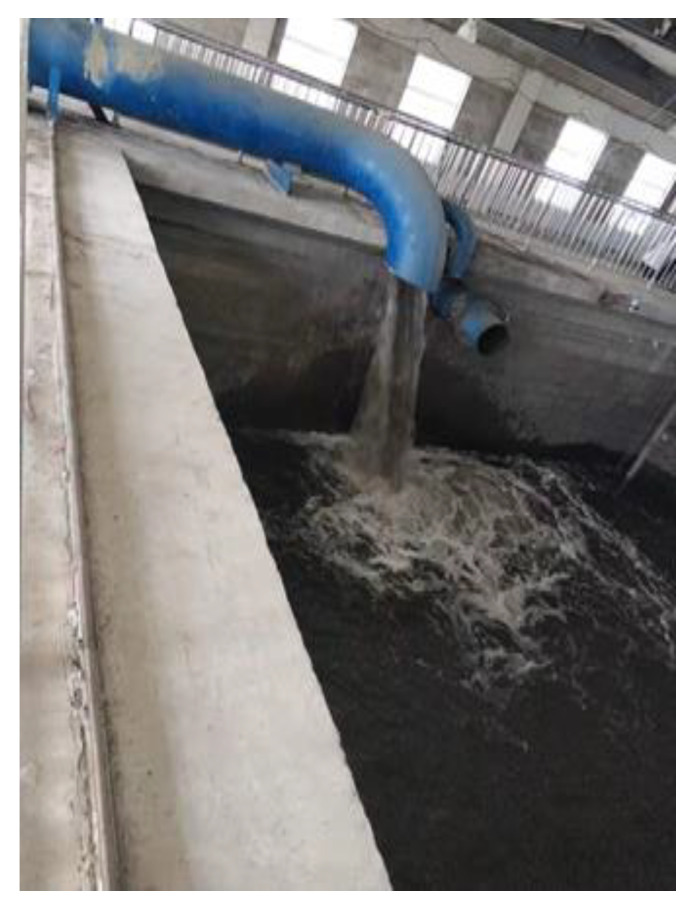
Mine water treatment tank.

**Figure 2:**
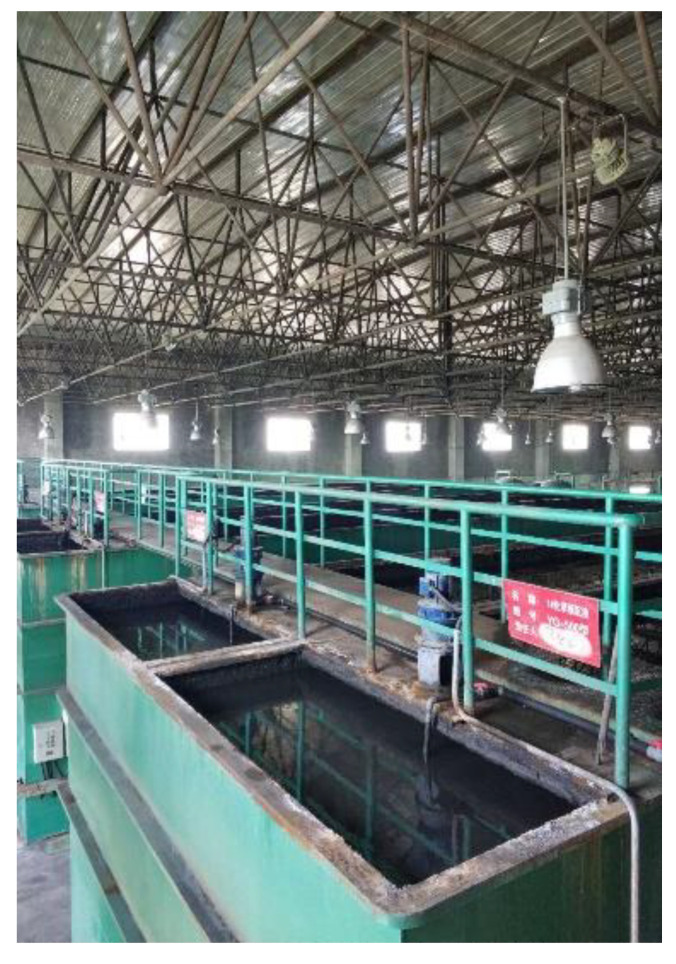
Mine water reuse reservoir.

Mine water undergoes a series of physical, chemical, and biochemical reactions during coal mining while in contact with coal strata, and it is influenced by human activities. This study used the Narim River No. 2 mine as the research location. The mine water treatment tank is shown in the Figure 1. The mining area is located in Ordos, Inner Mongolia, which is characterized by special geological conditions. The mine is situated in the basin of the Wuding River, which is the first tributary of the Yellow River, and has abundant water inflow. A previous investigation found that a large portion of the mine water is treated and reused within the area of the mine [1,2]. Furthermore, the treatment process is fixed and corresponding adjustments cannot be made according to changes in time or space. As a result, a large quantity of mine water is not treated in a timely manner and reuse efficiency is low. This not only leads to water resource loss, but also results in significant quantities of acid and organic pollutants in the mine water, thus causing serious damage to the mining environment [3,4,5,6]. In addition, the required quality of water used in the mine varies, such as for underground firefighting, grouting, and hydraulic support. The demand for production water must be met at all times, and the water quality requirements for domestic use and boiler water are more stringent. In addition, the lack of corresponding water supply points for different water directions further reduces the reuse efficiency of mine water. The mine water reuse reservoir is shown in the Figure 2. In summary, the reuse of mine water has low efficiency due to: (1) the large water inflow of the mine; (2) inefficiencies of the mine water treatment technology; and (3) the distribution of the mine water.

In view of the above problems, researchers have proposed a variety of solutions, such as underground water detection, mine water treatment automation, and combinations of mine water supply and drainage. Ref. [6] proposed calculation of groundwater reserves and their changes using gravity recovery, climate experiments, and a global hydrological model. They put forward an important basis for the detection of coal mine water inflow and the formulation of pretreatment scheme. Ref. [7] designed a control system using a fuzzy proportional controller to maintain biogas flow [7]. The controller function was developed in MATLAB software and embedded in a Nios II processor of FPGA. Ref. [8] designed a set of automatic monitoring systems for water resource purification [8], which was mainly aimed at the automatic control of water quality monitoring and chemical configuration. The sensor collects real-time data, and controls the starting and stopping of dispensing, sewage, and water pumps according to the control strategy. This system improves the automation and reuse efficiency of mine water treatment. Ref. [9] proposed an optimized combined system model to address the issues of the drainage, water supply, and environmental protection of a coal field using the Phillip multi-objective simplex method and fuzzy analytic hierarchy process [9]. The scheme improved the scope of the mine water treatment system and increased the reuse rate of mine water. Ref. [10] proposed a new optimization allocation tool for mining units of a drainage pipeline, aimed at minimizing hydrogen sulfide production in view of the rapid increase in water resource pressure in urbanization [10]. The network was simulated many times by combining the Monte Carlo method with SWMM. The method was applied to a sewage pipe network in Greece and ideal results were obtained.

To date, numerous studies on energy scheduling have been published [11,12]. Mine water is one of the main energy sources for mine production safety. An appropriate allocation of water resources can not only save water, but also significantly enhance mine production safety.

## 2. Scheduling Reuse System Model

### 2.1. Hierarchical Reuse Strategy

**Figure 3:**
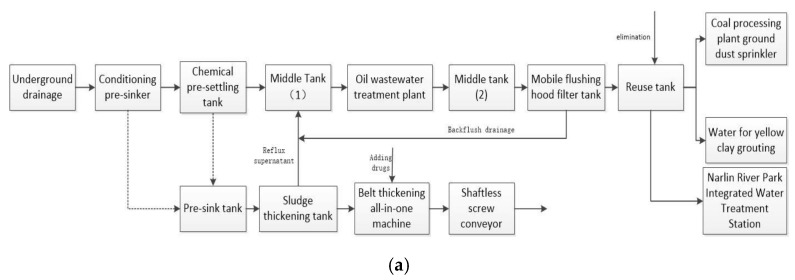

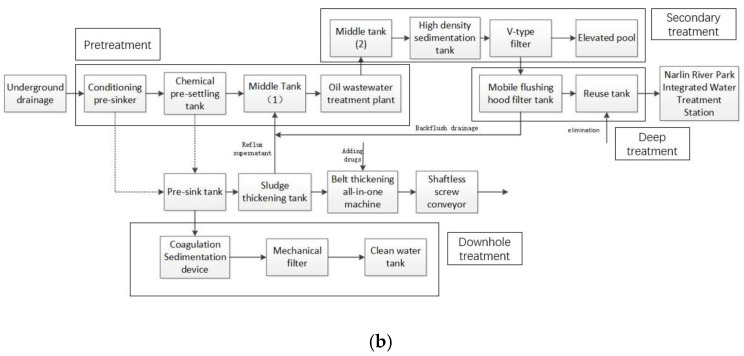
Process flow chart of mine water treatment: (**a**) traditional treatment process; (**b**) optimization of treatment reuse systems.

Significant differences exist in mine water treatment processes in different mining areas. This paper focuses on the treatment process of Narim River No. 2 mine, as shown in Figure 3a. The reclaimed water in the mining area is mine water following advanced treatment. This process requires a long period, which delays the reuse speed of mine water and indirectly affects the production of the mining area. Because the traditional mine water treatment and reuse system is simple and cannot undertake complex optimal dispatching for water reuse, this study aimed to improve the mine water treatment system. To increase the amount of underground mine water reuse, a coagulation sedimentation device and mechanical filter were added to treat the mine water. This increases the number of mine water reuse points of the underground clear water pool to meet the water quality requirements of underground water. Compared with the domestic water in the mining area, production water has the characteristics of large quantity, low water quality requirements, and concentrated water consumption points. Therefore, the reuse outlets in the pre-treatment, secondary treatment, and deep treatment stages were established to meet the requirements of the different water points in terms of water quality and quantity, as shown in Figure 3b.

The reuse rate and efficiency of mine water are the most direct means of reflecting the state of mine water reuse. Therefore, this study investigated the reuse water and treatment speed of the Narim River No. 2 mine, established the mathematical relationship among the participating quantities, and deduced the mathematical model of mine water optimal dispatching. The mathematical model was analyzed and solved using an improved particle swarm optimization algorithm, and the optimal allocation scheme of mine water treatment was calculated.

**Figure 4:**
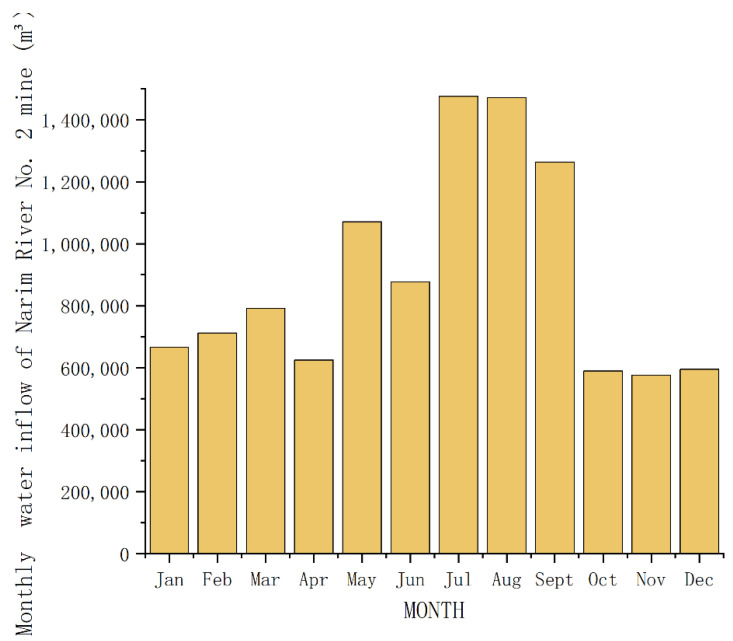
Monthly water inflow of Narim River No. 2 mine.

The mine water dispatching system comprises underground dispatching and ground dispatching. Based on a survey of underground and ground water consumption, the monthly water consumption in the Narim River mine in 2015 is shown in Figure 4; January, February, March, and December constitute the heating season, and May, June, July, and August constitute the non-heating season. The survey results of water quality and water quantity at the water use point of the mining area are shown in Table 1. Surface water consumption accounts for about 73.4% of the water consumption in the mining area, including production water used for ground dust removal, firefighting, coal preparation, heat exchange stations, and cooling, in addition to domestic water used for drinking, greening, and boilers in the mining area. The water consumption points are divided according to the water quality conditions. The water supply points are mainly distributed between the middle, high-level, and reuse tanks. Underground water points include those for underground firefighting, grouting, hydraulic support, cooling, and underground dust removal. The water supply point is the underground clean water tank.

The water quality and quantity of the mine water treatment process vary at different stages. The underground surface water consumption points are matched with the treatment process reuse pool. On this basis, combined with the priority order of water consumption points, the variation ranges of the water consumption points are preset. The preset variation results are shown in Table 1. Based on a field investigation of mine water treatment capacity and subsequent analysis, the mine water treatment speeds of all levels of the water consumption points are shown in Table 2. Because the actual treatment process has not changed, the fastest treatment speed is adopted in the experimental simulation; that is, the mine water treatment speed during the non-heating season.

### 2.2. Reuse Strategy Model

The ultimate goal of the mine water optimal dispatching system is to adjust the operation scheme of the system. Under the conditions of meeting the water quality and quantity restrictions of the mining area, the mine water after treatment is optimally distributed, so that the reuse rate of mine water and the working efficiency of the treatment system are improved. In this study, the lowest reciprocal of the sum of mine water reuse and its treatment time was taken as the objective function, and the water quality and quantity of each water consumption point was taken as the constraint condition. For a certain period of time *t*, the mathematical model of system optimal scheduling was established

(1) $$\min fit=\overset{M}{\underset{i=1}{\sum }}\left(\frac{1}{\omega_{1}\frac{C_{i}-Q_{i}}{S_{i}-Q_{i}}+\omega_{2}\frac{t_{imax}-t_{ic}}{t_{imax}-t_{imin}}}\right)$$

where $S_{i}$ is the maximum amount of mine water recycling, $C_{i}$ is the recycling amount of the ith water consumption point, $Q_{i}$. is the mine water reuse amount of the ith water used in the original system, $t_{imax}$ is the time used to treat the maximum reuse amount of mine water, $t_{imin}$ is the minimum time used to treat the minimum amount of mine water recycling, $t_{ic}$ is the time used by the ith water point to reuse mine water. $\omega_{1}$, $\omega_{2}$ are the weight coefficients, which are 0.6 and 0.4, respectively.

### 2.3. Reuse System Constraints

(1) The balance of water supply and demand in the mining area. During any period of time, the mine water inflow into the mine water treatment system should be equal to the sum of water consumption and discharge of each water point. Because evaporation and loss of water in the treatment process are inevitable, they are ignored in this model.

(2) $$S=\sum_{i=1}^{M}C_{i}+D$$

where *S* is the total water inflow, $C_{i}$ is the water consumption of each water point, and D is the mine water discharged after treatment.

(2) Water supply capacity of mine water treatment at all levels. At each stage of the treatment system, the amount of water to be used in the treatment tank is limited. During the operation of the mine water treatment system, it is necessary to establish multiple reuse tanks at each treatment level to satisfy the water resource utilization of the mining area without delaying the normal operation of the treatment system. Due to space constraints, the size of the reservoir needs to be limited according to the site of the mining area. Therefore, in the process of scheduling and reuse, it should be first determined whether the water quantity in the reuse tank can meet the demand of water consumption, and whether to continue scheduling.

(3) $$B_{min}\leq B_{i}\leq B_{max}$$

where $B_{i}$ is the water supply of the I treatment stage, $B_{min}$ is the minimum water supply in stage I, $B_{max}$ is the maximum water supply in stage *i*.

(3) The water quality condition of equipment in the mining area. There is an upper limit for each level of water treatment. Because the mine water contains a variety of minerals and is characterized by an acid–base imbalance, a variety of chemicals are required for treatment, and each level of water quality treatment is subject to a different water quality standard.

(4) $$Z_{imin}<Z_{i}<Z_{imax}$$

where $Z_{i}$ is the water quality of treatment stage *i*,$\;Z_{imin}$ is the minimum standard of water quality in stage *i*, $Z_{imax}$ represents the highest water quality standard of stage *i* treatment.

(4) Mine water treatment speed. Although mine water treatment operates continuously, in practice the size of the water reuse tank is limited, and a certain amount of time is required for storage, particularly for the process of dosing sedimentation, which takes a long time.

(5) $$V_{imin}<V_{i}<V_{imax}$$

where $V_{i}\;$is the purification rate of mine water in stage *i*.$\;V_{imax}$ represents the fastest treatment speed of the *i*th treatment stage, $V_{imin}$ is the minimum treatment speed of stage *i*.

Based on the above conclusions, this study derived four constraints for the mine water optimal operation model, namely, one equality constraint and three nonlinear constraints. Therefore, the optimal scheduling model proposed in this paper is an optimization problem with nonlinear constraints. For this kind of problem, by introducing penalty function, the optimal scheduling problem can be transformed into an unconstrained optimization problem and then solved. In general, the above constraints are converted into the calculation of penalty function. For the calculation of penalty value, refer to the formula

(6) $$\varphi =\sum_{n=1}^{N}{\left(\max\left\{0,-u_{n}(\vec{x})\right\}\right)}^{2}+\sum_{h=1}^{H}\left({\left|z_{h}(\vec{x})\right|}^{2}\right)$$

where φ indicates the penalty value, *N* is the number of inequality constraints in the optimal scheduling problem, *H* is the number of equality constraints, $u_{n}\left(\vec{x}\right)\;$is the result of the transformation of the *n*th inequality constraint,$\;z_{h}\left(\vec{x}\right)$ is the result of the transformation of *H* equality constraints

(7) $$u_{n}(\vec{x})\geq 0$$

For *h* equality constraints in the optimization problem, $z_{h}\left(\vec{x}\right)\;$is the converted form, and the conversion method is

(8) $$z_{h}(\vec{x})=0$$

Regarding the calculation of the penalty value, Equations (7) and (8) show that if the variable exceeds the limit given by the inequality constraints, the penalty value is $|-u_{n}\left(\vec{x}\right){|}^{2}$, otherwise it is 0. If a variable exceeds the limit given by the equality constraint, the penalty value is ${\left|z_{h}\left(\vec{x}\right)\right|}^{2}$; otherwise, it is 0. Thus, a large positive integer δ can be multiplied with the penalty value, which is then included in the objective function proposed in this paper. Because the aim of the optimal objective function is to find its minimum value, a penalty value can be added to form an augmented function relative to the original objective function

(9) fin(f)=f+δφ

where fin(f) indicates the final objective function, f is the objective function, δ is a positive integer with a value of 10^6^, φ indicates the penalty value.

As can be seen from the above formula, due to the magnitude of the value of δ, when the variables exceed the constraints proposed in this paper, the result of the objective function becomes very large. Thus, the objective can be quickly determined as a non-optimal solution.

## 3. Design and Optimization of Mine Water Dispatching Method Based on Particle Swarm Optimization

The particle swarm algorithm simulates the feeding process of birds by setting each bird involved in the feeding behavior as a particle with no mass and volume [13,14,15]. In the basic particle swarm algorithm, each particle is treated as a massless, volume-free particle in the search space. Suppose the dimension of the search space is D and the number of the target population is n. The *i*th particle in the population space can be represented as a position in the D-dimensional space, expressed as $X_{id}=\left[x_{i1},x_{i2},x_{i3}\ldots x_{iD}\right]$,(d=1,2,3…D). If Xi is substituted into the objective function, its fitness value can be obtained, and the superiority or inferiority of the obtained result can then be judged by comparing the magnitude of its fitness value. Another important parameter in the iteration of the algorithm is the flight speed of the particle $V_{id}=\left[v_{i1},v_{i2},v_{i3}\ldots v_{iD}\right]$(d=1,2,3…D), which denotes the flight speed of the i(i=1,2,3…n)th dimension of the first particle. In the search space range, assuming that the current best position found by the *i*th particle is Pbest_id_ = (p_1d_, p_2d_, p_3d_, ......, p_nd_,), the best position identified by the population in the search space range can be expressed as Gbest_gd_(G_1d_, G_2d_, G_3d_, ......, G_gd_).

The formula of each update iteration is

(10) $$V_{id}^{k+1}=\omega V_{id}^{k+1}+C_{1}\varepsilon \left(P_{\mathrm{best}\;}^{k}-x_{id}^{k}\right)+C_{2}\mu \left(G_{\mathrm{best}\;}^{k}-x_{id}^{k}\right)$$

(11) $$X_{id}^{k+1}=X_{id}^{k}+V_{id}^{k+1}\ldots d=1,2,3\ldots D$$

*C*_1_ represents the weight coefficient of the optimal value searched in the historical search, which is the recognition of the particle itself. *C*_2_ represents the weight coefficient of the optimal value identified by the particle swarm in the search. This is the recognition of the population in the cluster, which is usually 2. Variables ε and μ are random numbers distributed in the interval [0, 1]. The individual extreme pbest and the global extreme Gbest are expressed by Equations (12) and (13), respectively.

(12) $${\;\mathrm{Pbest}\;}_{i}(k)=\mathrm{argmin}\left\{fit\left(X_{i}(1)\right),\;fit\;\left(X_{i}(2)\right),fit\left(X_{i}(3)\right),\ldots ,fit\left(X_{i}(k)\right)\right\}$$

(13) $$\mathrm{Gbest}(k)=\mathrm{argmin}\left\{{\mathrm{Pbest}}_{1}(k),{\;\mathrm{Pbest}\;}_{2}(k),{\;\mathrm{Pbest}\;}_{3}(k),\ldots ,{\;\mathrm{Pbest}\;}_{4}(k)\right\}$$

ω represents the weight coefficient of the particle [16,17,18], also known as the inertia factor, which is a linearly decreasing variation parameter. The specific formula is

(14) $$\omega_{k}=\omega_{max}-\left(\frac{\omega_{max}-\omega_{min}}{K_{max}}k\right)$$

Here, $\omega_{max}$ = 0.9, $\omega_{min}$ = 0.4, $K_{max}$ is the maximum number of iterations, and *k* is the current number of iterations.

With an increase in the iteration number *k*, the velocity and position of particles in the population are constantly changing. In addition, $P_{best}^{k}-x_{id}^{k}$ is called self cognition and $G_{best}^{k}-x_{id}^{k}\;$is called social cognition [19,20,21,22,23].

Particle swarm optimization has been widely used in many basic science and application fields. In the field of artificial intelligence, it is most often used in the training of neural network models in artificial intelligence; in electrical engineering, it can be used to control product or power costs. In this study, particle swarm optimization (PSO) was used to address the problem of mine water optimal scheduling.

**Figure 5:**
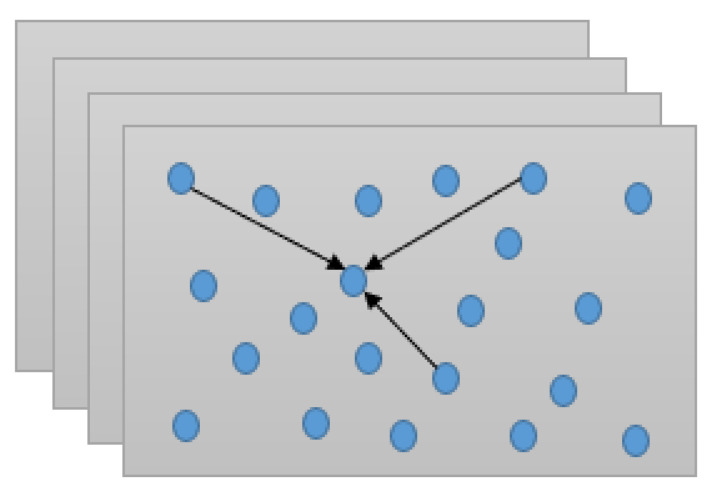
Particle swarm optimization for graph optimization.

According to the characteristics of particle swarm optimization, each particle is composed of multidimensional space. In the iterative process, the parameters in each bit space are moving to the optimal position, which is the process of particle optimization, as shown in Figure 5.

The aim of the mine water optimal scheduling model established in this paper is to optimize the total scheduling amount of multiple objective reuse tanks. The objective of optimization is to ensure the water in each reuse tank is fully utilized under the premise of meeting the constraints, and to obtain the optimal solution of the objective function established in this paper.

According to the characteristics of the particle swarm optimization algorithm and the goal of mine water optimal scheduling and reuse, this paper combines each scheduling tank with the spatial dimension of particles. The optimization process of particles in the spatial dimension corresponds to the scheduling and optimization process of the mine water reuse system. The common goal is to make the objective function f(x) obtain the optimal value.

**Figure 6:**
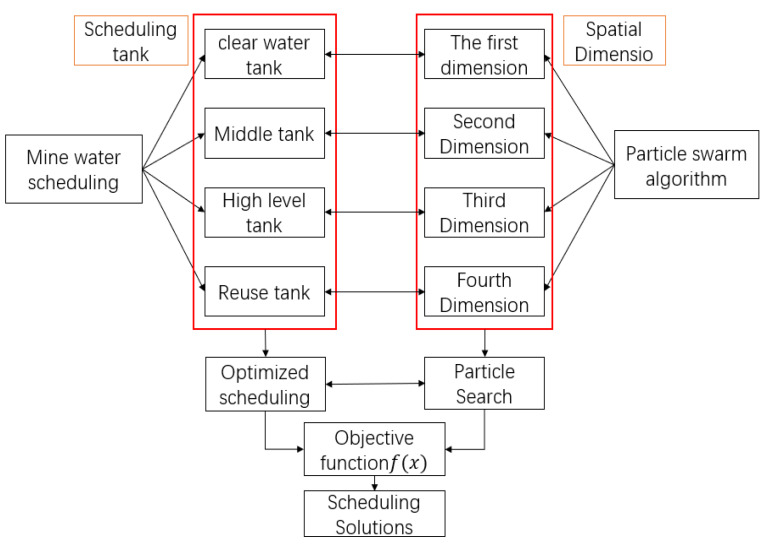
Combined graph of mine water dispatching and particle swarm optimization algorithm.

Figure 6 shows that mine water dispatching is mainly used in the clean water, intermediate, high water, and reuse tanks. The iterative optimization of particle swarm optimization is carried out in the multi-dimensional space of particles. Therefore, the spatial dimension of particles is set to four-dimensional, and different scheduling schemes are found in each iteration calculation. The particle swarm optimization algorithm calculates the target function according to the scheme until the termination condition of iteration is reached or the particle finds the best advantage; that is, when the objective function obtains the minimum value, the algorithm stops iterating and outputs the calculation results. Thus, the mine water scheduling scheme can be obtained.

## 4. Inertia Weight Strategy Analysis

### 4.1. Inertia Weight Decreasing Strategy

Inertia weight is one of the main factors that affect the effect of particle swarm optimization. The larger the inertia weight, the stronger the ability of global space search. When the inertia weight factor ω is small, the local search ability of the particle will be enhanced, and it will be close to the optimal value. The disadvantage is that lower weight will reduce the optimization speed of particles.

**Figure 7:**
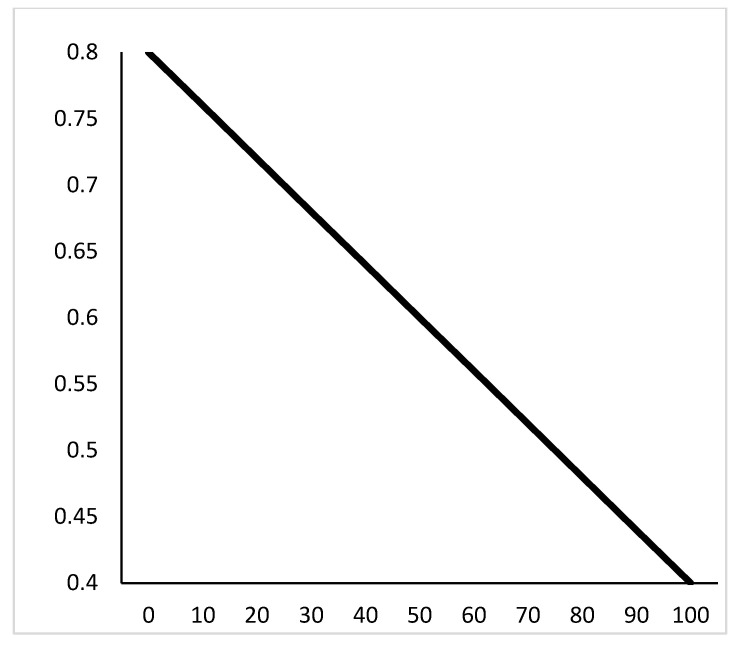
Linear decreasing function.

In the standard case, the inertia weight is a linear decreasing function. The linear decrement formula is shown in Equation (14). The formula is shown in Figure 7.

Figure 7 shows that inertia weight is a linear function of the number of iterations. When the initial iteration starts, the inertia weight ω is relatively large. we can see that the particle velocity is relatively large in the initial iteration from formula 10, which has a good global search ability, but the local search ability is weak. With the accumulation of the number of iterations, the value of ω is smaller and smaller, and the search speed of particles is smaller and smaller. However, the search is more detailed, which can find the best point in the local range. However, if the best point is not met at the beginning, the subsequent iterative optimization will be affected by the deviation.

There is also a differential decreasing inertia weight, and the specific calculation formula is

(15) $$\omega =\omega_{max}-\frac{\omega_{max}-\omega_{min}}{T_{\max}{}^{2}}t^{2}$$

In the formula $\omega_{max}$ is the maximum and initial value of inertia factor, $\omega_{min}$ is the minimum and the final value, $t_{max}$ is the maximum number of iterations, t is the current number of iterations.

**Figure 8:**
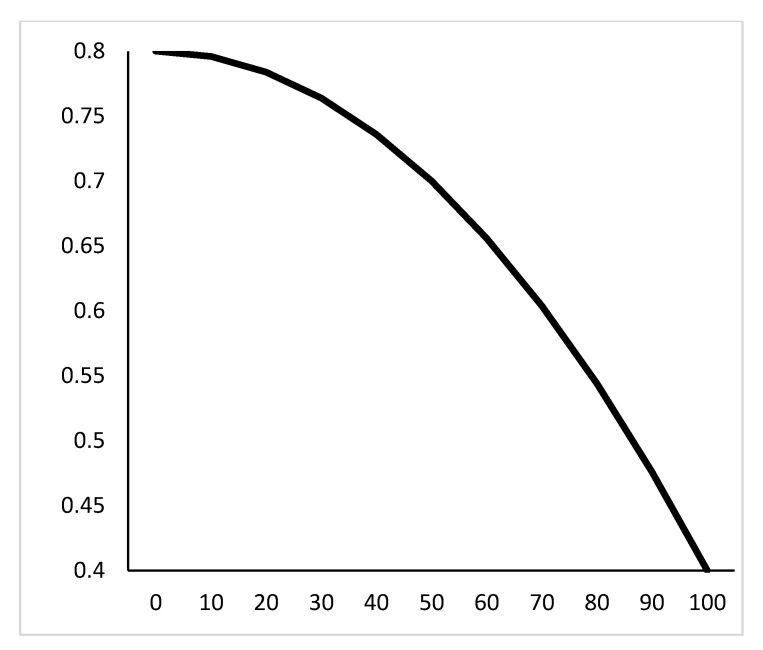
Exponential decreasing function.

Figure 8 shows that, the inertia weight ω is also negatively correlated to the number of iterations, and inertia weight is a quadratic function of the number of iterations. This function is an exponential declining function. In the initial state, particle swarm optimization algorithm will search for more optimal values in global space. With the iteration, the possibility of finding a global optimal will be greatly increased.

### 4.2. Improvement Strategy of Inertia Weight

**Figure 9:**
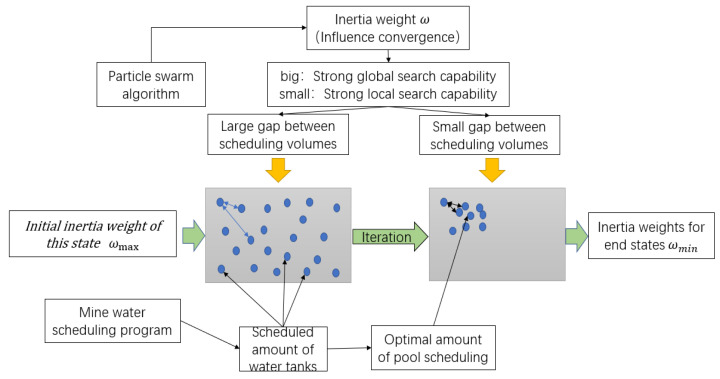
Adaptive adjustment chart of mine water dispatching weight.

The difference between adaptive particle swarm optimization and basic particle swarm optimization in solving mine water scheduling problem is the improvement of inertia weight. For example, the influence of inertia weight proposed in the improved scheme on the convergence of PSO is that the global search ability is strong when the inertia weight is large, and the local search ability is strong when the inertia weight is small [24]. According to the goal of optimal operation of mine water, combined with the convergence characteristics of particle swarm optimization, the inertia weight ω is calculated. The improvements shown in Figure 9 are made.

Figure 9 shows that, in the initial state, the inertia weight of the particle swarm optimization algorithm is the maximum state and, at this time, the scheduling quantity of each tank in the schedulable scheme is in a random state, which corresponds to the large Euclidean space distance between the particle dimensions. Therefore, this paper relates the inertia weight, which affects the convergence state of the algorithm, to the Euclidean space distance of the particle dimension in the particle swarm optimization algorithm. The improved scheme of inertia weight proposed in this paper was examined. Under continuous iteration, the scheduling gap between each dimension decreases, and the value of the inertia weight also decreased, which enhances the local search ability of the PSO.

The improvement of the particle swarm optimization algorithm proposed in this paper not only gradually decreases the weight with the iterative optimization process, and adapts to the change of state, but also limits the size of the inertia weight in terms of spatial distance. Thus, the inertia weight changes adaptively within the specified range, meeting the needs of the particle swarm optimization algorithm for mine water optimal scheduling.

In the basic particle swarm optimization, the linear inertia weight change causes the iteration of the algorithm to fall into a local optimum too early, which leads to an imbalance in local and global search ability. Therefore, this paper dynamically adjusts the inertia weight in the iterative process through the position of all particles in the population; with the increase in the number of iterations, all particles move towards the optimal value, and the inertia weight should be appropriately reduced, so as to improve the local search [25,26] and reduce the number of iterations. In each iteration, the Euclidean distance between particles in each dimension can be calculated as

(16) $$s_{k}=\sum_{i=1}^{N}\sum_{j=i}^{N}\sqrt{\sum_{k=1}^{D}{\left(x_{i}^{k}-x_{j}^{k}\right)}^{2}}$$

where *k* is the dimension, *D* is the maximum spatial dimension, n is the number of examples, $s_{k}$ is the sum of the distances of the particles in the kth dimension, $x_{i}^{k}$ is the position of the ith particle in the kth dimension.

By comparison, the sum of the distances between the particles of each dimension is obtained, and the maximum, minimum, and average of the distances are obtained.

(17) $$s_{ave\;}=\frac{1}{N-1}\sum_{K=1}^{D}s_{k}$$

According to the calculated distance between particles, the evolution factor F is calculated

(18) $$f_{i}=\frac{S_{ave\;}-S_{min}}{S_{max}-S_{min}}\in \left[0,\;1\right]\ldots i=1,2,3\ldots N$$

where $f_{i}$ is the evolutionary factor,$\;s_{max},s_{min,}s_{ave}$ represents the maximum, minimum, and average distance, respectively, between particles in each dimension.

ω does not simply decrease over time, but should change with the evolution state.Previous research has found that, if the PSO is in a reasonable operation state,$\;\omega_{d}$∈[0.4, 0.95]. This article selects the same change range. Because the change range of f is 0–1, the evolution factors should have the relationship

(19) $$\omega_{d}=\frac{1}{1+1.5e^{-3.35fi}}\in [0.4,0.95]$$

where $\omega_{d}\;$is the inertia weight and the initialization setting is 0.95. In the initial stage of the algorithm, *f* and *ω* are larger. In contrast, in the late convergence state, f and ω are smaller. It is more advantageous for the local search to reduce the search time.

Therefore, the new formula for each iteration is

(20) $$\omega_{d}=\frac{1}{1+1.5e^{-3.35fi}}$$

(21) $$X_{id}^{k+1}=X_{id}^{k}+V_{id}^{k+1}\ldots d=1,2,3\ldots D$$

(22) $$V_{id}^{k+1}=\omega_{d}V_{id}^{k+1}+C_{1}\varepsilon \left(P_{\mathrm{best}\;}^{k}-x_{id}^{k}\right)+C_{2}\mu \left(G_{\mathrm{best}\;}^{k}-x_{id}^{k}\right)$$

where $\omega_{d}$ is the weight coefficient of the improved algorithm, *X* is the position of the particle, *V* is the velocity of the particle, *k* is the iteration number, *C*_1_ and *C*_2_ are cognitive coefficients, and the value is 2. ε,μ are random numbers between 0 and 1.

When solving the problem, the water demand of the mining area is taken as the input of the model, and the scheduling strategy of mine water is taken as the output of the model:

(1) The state, population size, spatial dimension, iteration times, and parameters of each water supply node are initialized.
(2) The fitness value pi, individual extreme value pi_best_ and global extreme value G_best_ of each particle are calculated. If pi < pi_best_ is satisfied, then pi_best_ = pi; if pi < G_best_, then G_best_ = pi.
(3) The inertia weight of the improved algorithm is updated.
(4) The position and velocity of each iteration particle is updated.
(5) Judge whether the particle reaches the termination condition. If it meets the condition, terminate the search. If not, return to the second step and continue.

**Figure 10:**
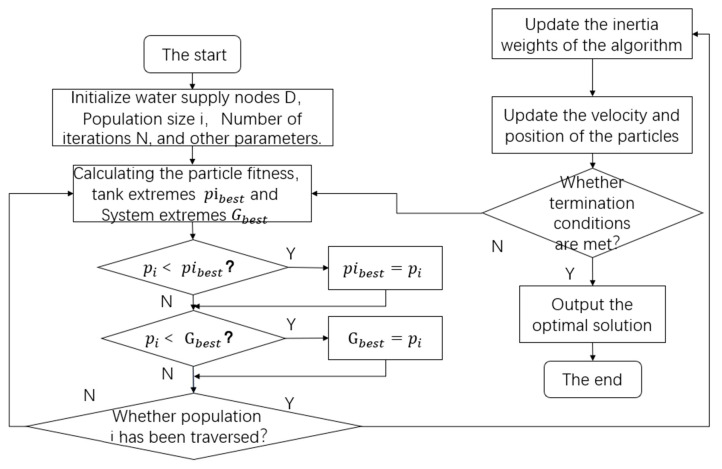
Flow chart of scheduling system optimization based on particle swarm optimization.

To more clearly reflect the operation principle of the improved particle swarm optimization algorithm in the mine water optimal scheduling system, a flow chart of mine water optimal scheduling based on the improved particle swarm optimization algorithm, which illustrates the above process, is shown in Figure 10.

### 4.3. Test Results and Analysis

To verify the convergence accuracy and speed of the improved algorithm, this study simulated the improved adaptive weight particle swarm optimization algorithm, using four classic test functions, as shown in Table 3. The particle swarm optimization (PSO) with linear weight-decreasing (LDIW-PSO), exponential weight-decreasing (EDIW-PSO), and adaptive weight-decreasing (ADIW-PSO) inertia weight strategies were simulated respectively; the optimal values [27,28,29] were calculated and the performance of the three optimization algorithms was compared.

To improve the convergence speed and accuracy [30,31,32,33] of the algorithm, the number of particle swarm optimizations was 50 and the maximum number of iterations was 100. The test functions are presented in Table 3.

To more clearly and intuitively present the improved PSO face thinning effect, the convergence of the four test functions was analyzed by comparing three different particle swarm optimization algorithms, namely LDIW-PSO, EDIW-PSO, and ADIW-PSO. The convergence results are shown in Table 4.

**Figure 11:**
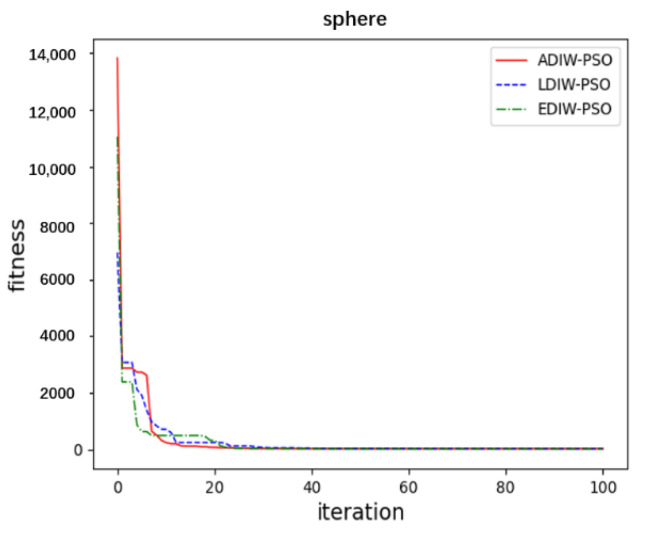
Sphere function.

**Figure 12:**
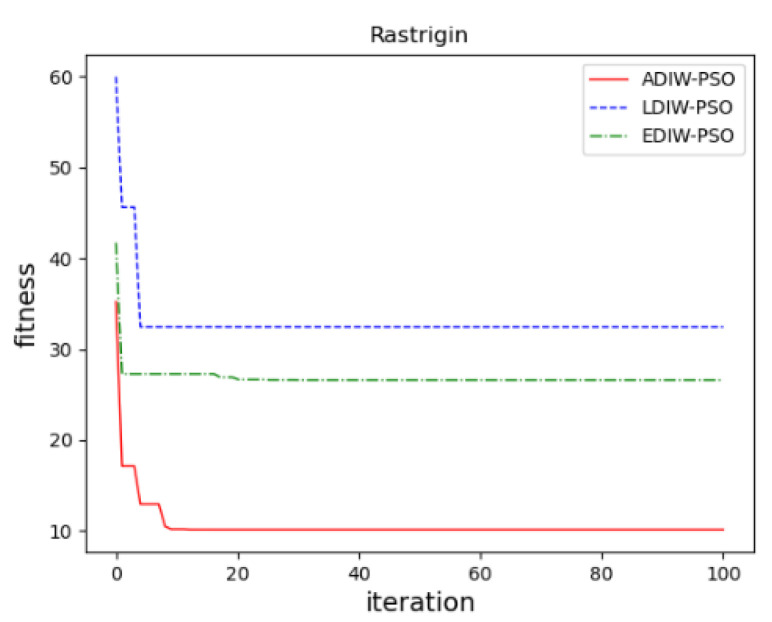
Rastrigin function.

**Figure 13:**
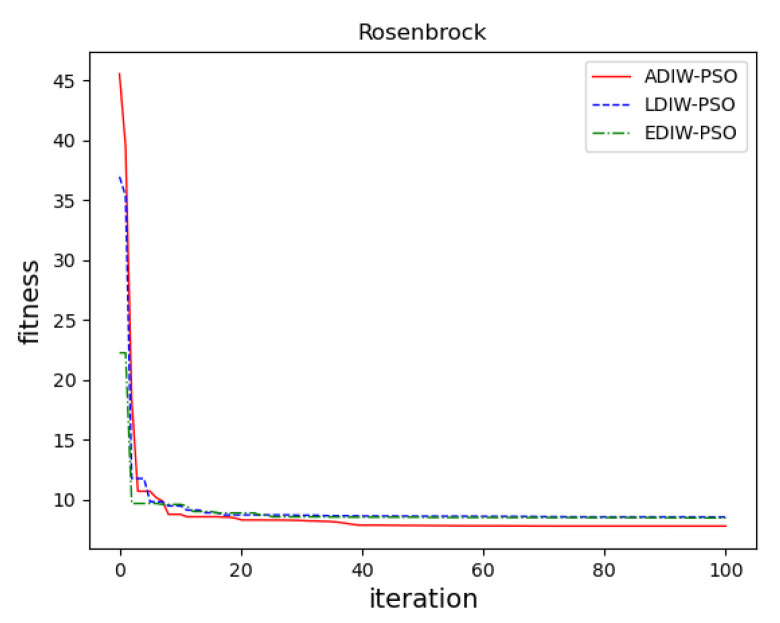
Rosenbrock function.

**Figure 14:**
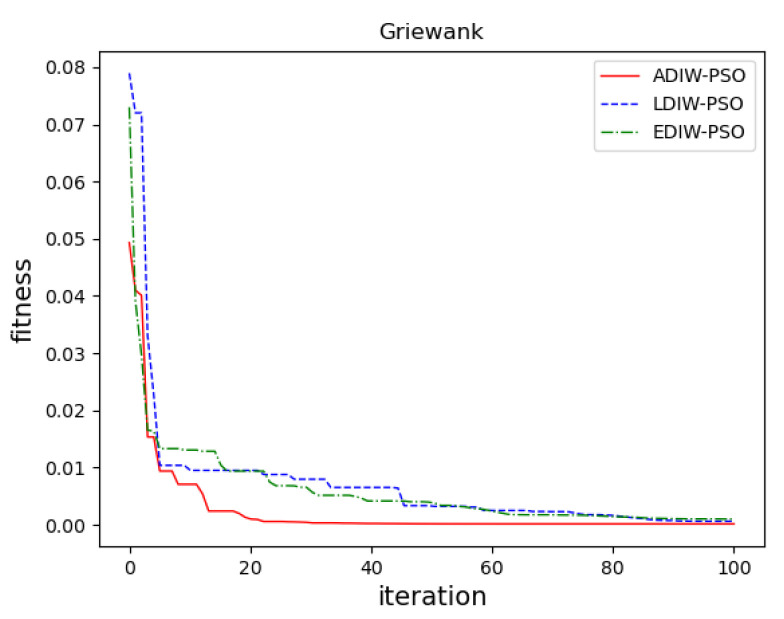
Griewank function.

In sphere function, Figure 11 show that, the convergence accuracy of three algorithms is close. ADIW-PSO achieves the optimal value about 10 iterations. LDIW-PSO and EDIW-PSO need about 22 iterations to achieve the optimal value. which shows that ADIW-PSO is faster in global search. For the Rastrigin function, Figure 12 show that ADIW-PSO is better than the other two algorithms in convergence accuracy. Which shows that ADIW-PSO is more practical. For Rosenbrock function, Figure 13 show that the convergence accuracy and speed of the three algorithms are close. These indicating that there is little difference in local search ability. In the Griewank function, Figure 14 shows that ADIW-PSO is obviously better than the other two algorithms in search speed. Which indicates that it can quickly jump out of the local search limit. In summary, the results show that the adaptive weight-decreasing particle swarm algorithm proposed in this paper can effectively improve the optimization accuracy and speed of the algorithm.

## 5. Simulation Examples and Experiments

### 5.1. Example of Results Validation

The scheduling direction of the mine water scheduling system has two major components: underground scheduling and surface scheduling. Based on an investigation of the water consumption of underground and surface water, the monthly water surges in the Narim River mine area in 2015 are shown in Figure 4, in which January, February, March, and December constitute the heating season, and May, June, July, and August constitute the non-heating season. The water quantity survey results of the water points in the mine area are shown in Table 5. Surface water consumption accounts for about 73.4% of the water consumption in the mine area, including production water for ground dust removal water, firefighting, coal preparation, heat exchange stations, and cooling; domestic water is used for drinking, greening, and boilers in the mine area. The water points are divided according to the water quality conditions, and the water supply points are mainly distributed in the intermediate and high ponds. The water supply points are mainly located in the intermediate, high level, and reuse tanks; the underground water supply points include water for underground firefighting, grouting, hydraulic support, cooling, and underground dust removal. The water supply point is the underground clear water tank. According to the different water quality requirements of the water points in the mine area, the scheduling method of grading and dividing the quality of mine water supply is adopted to achieve reasonable distribution of mine water, to maximize the mine water resources and make the mine water treatment process more efficient.

To improve the convergence speed and accuracy of the PSO, combined with the actual variable conditions of the mine water, this paper set ion beam to 100, dimension to 4, and maximum number of update iterations to 100. Equations (1)–(9) were applied to the mine water in the 2015 heating and non-heating seasons for the water influx to determine the best calculation. Python simulation software was used for comparison, and simulation results are as follows.

**Figure 15:**
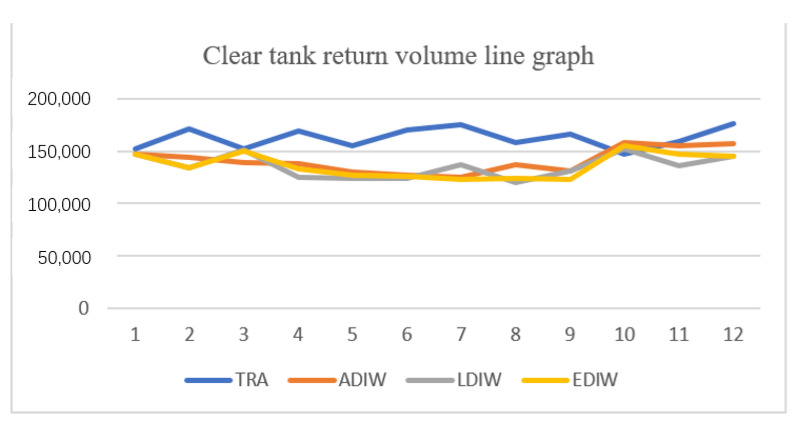
Distribution of mine water reuse in clear water tank.

**Figure 16:**
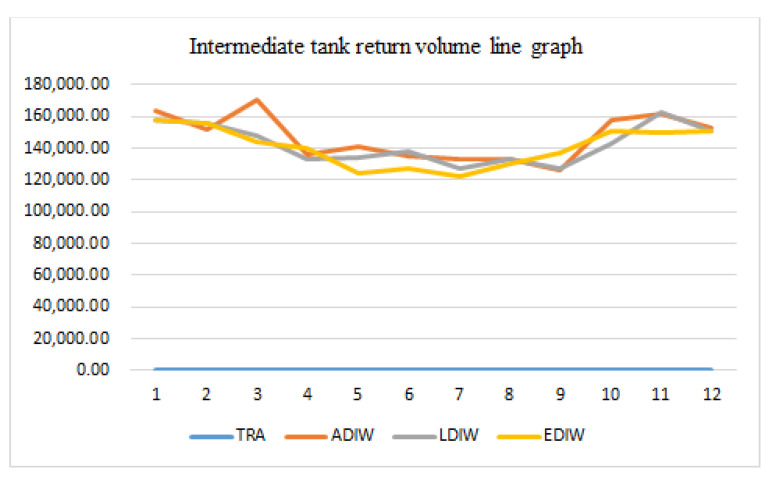
Distribution of mine water reuse in middle tank.

**Figure 17:**
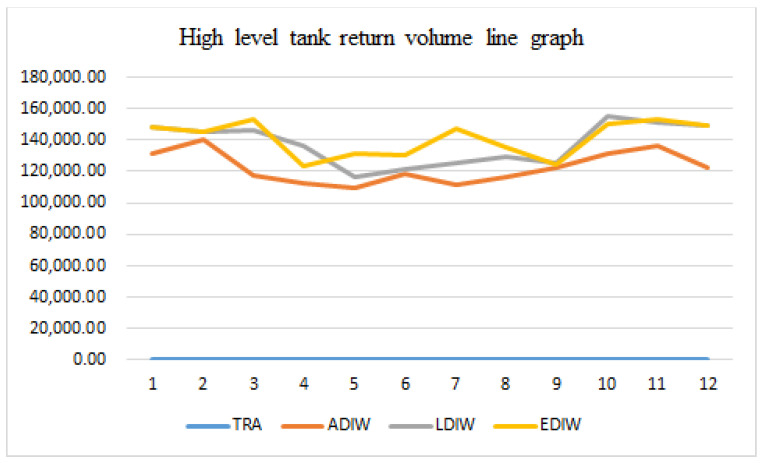
Distribution of mine water reuse in high level tank.

**Figure 18:**
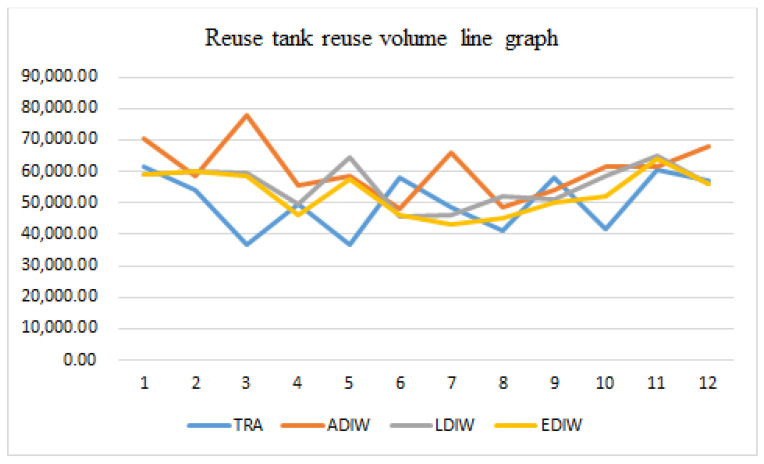
Distribution of mine water reuse in reuse tank.

Figure 15, Figure 16, Figure 17 and Figure 18 show that the traditional mine water dispatching approach is simpler, with no middle or high-level tanks, and dispatched water is zero. Under different optimization systems, the dispatch of mine water is redistributed, with the high and middle tanks sharing most of the surface mine water dispatching tasks. In addition, the reuse tanks have stricter water quality for a longer number of reuse time periods, and have relatively small reuse volumes.

By optimizing the statistical analysis of the reuse volume of mine water at each reuse point, a comparison of the volume under the traditional mine water reuse mode and the optimized scheduling system mode was undertaken, which also includes the specific allocation volume for the heating and non-heating seasons. The results are shown in Table 6.

After optimizing the calculation of water consumption for the Narim River No. 2 mine, it can be seen by comparison that the reuse amount during the heating season under the traditional model is mainly reused by the clear water and reuse ponds, and the reuse amount is limited. Using the PSO and the improved system for deployment, the reuse amount of the system increased significantly and was mainly concentrated in the middle and high tanks. To verify the practicality of the algorithm for mine water scheduling and to compare the effectiveness of the improved adaptive PSO more clearly, the reuse rate and reuse time were compared, as follows.

Mine water reuse ratio. In the case in which only the reuse of mine influx water is considered, the ratio of the reuse amount of mine influx water is

(23) $$\mu_{1}=\frac{\sum_{i=1}^{N}C_{i}}{S}$$

where *N* indicates the mine water reuse point at all levels, $C_{i}$ indicates the mine water reuse amount at all levels of reuse points, and *S* indicates the mine water surge volume. Here it is assumed that the mine water in the treatment has no other reuse than the flows in and out.

Mine water reuse treatment time. The treatment rate of each level of the investigated mine water treatment system was used to calculate the overall system treatment reuse operation time

(24) $$t_{n}=\frac{C_{i}}{V_{i}}\cdots i=1,2\ldots N$$

where $t_{n}$ indicates the treatment time of mine reuse water at the *n*th reuse level, $C_{i}$ indicates the amount of mine water reused at each reuse point, and $V_{i}$ indicates the treatment rate of mine reuse water at each reuse point. Mine water reuse treatment time is taken as the maximum value calculated at each level.

Mine water reuse rate. That is, the amount of mine water reuse during the same time period, under different treatment reuse processes or different algorithm calculations

(25) $$v=\frac{\sum_{i=1}^{N}C_{i}}{t_{nmax}}$$

where v indicates the reuse rate of mine water, *N* indicates the reuse points of mine water at all levels, $C_{i}$ indicates the reuse amount of mine water at all levels, and $t_{nmax}$ indicates the maximum treatment time of mine reuse water.

According to the reuse rate of mine water in Table 6 and the Equations (23)–(25), the data after optimization of the algorithm were compared with the system before optimization; the calculation results are shown in Table 7.

As can be seen from Table 7, during the heating season, due to the multi-target reuse in the mine, the optimized system improved the reuse rate by 46.2%, which is significantly higher than that of the traditional mine water reuse method. The reuse time was reduced by 147.59, 99.82, and 104.79 h, respectively, compared with the traditional scheduling method, and the reuse rate was improved by 634.19, 562.6, and 569.53 m/h, respectively. In the same scheduling test, the adaptive weight particle swarm algorithm had a lower scheduling time compared with the linear weight and nonlinear weight particle swarm algorithms. Furthermore, the reuse operation of the system was faster, which verifies the practicality of the algorithm in the process of mine water scheduling.

As can be seen in Table 8, the mine water reuse rate of mine water increased by 17.5% in the non-heating season, which is relatively small, due to the high influx of water from the Narim River No. 2 mine and the limited water use at the mine site. The treatment reuse time of mine water was reduced by 117.51, 53.51, and 61.18 h, and the reuse rate was reduced by 414.86, 345.73, and 353.32 m/h, respectively, which verified the feasibility and practicality of the system and the algorithm.

Table 8 shows that the reuse rate significantly improved, indicating that the overall operating efficiency of the system increased. Thus, more mine water is able to be treated at one time, effectively reducing the risk caused by the high water consumption of mine water. Therefore, the results of the real data analysis of the Narim River show that the scheduling model and the improved algorithm method proposed in this paper can effectively address the problem commonly faced in mines. Based on the analysis of the original data of the Narim River, the effectiveness and feasibility of the scheduling model and the improved algorithm proposed in this paper are therefore proven.

### 5.2. Discussion

The control system of the intelligent optimal dispatch of mine water is the core of a mine water treatment system. This system has the ability to analyze, process, and predict the state of the large quantity of data collected by the sensing system [34]. Furthermore, the system can perceive the reuse environment and status of mine water using a variety of sensors, and obtain processing information at all levels of the mine water treatment system. Combined with the above-mentioned particle swarm algorithm, the optimized scheduling model mentioned in this article can be integrated into the control system to make predictions regarding the quantity and quality of water resources in the mining area. The system rationally allocates the underground–surface mine water to co-ordinate its scheduling.

**Figure 19:**
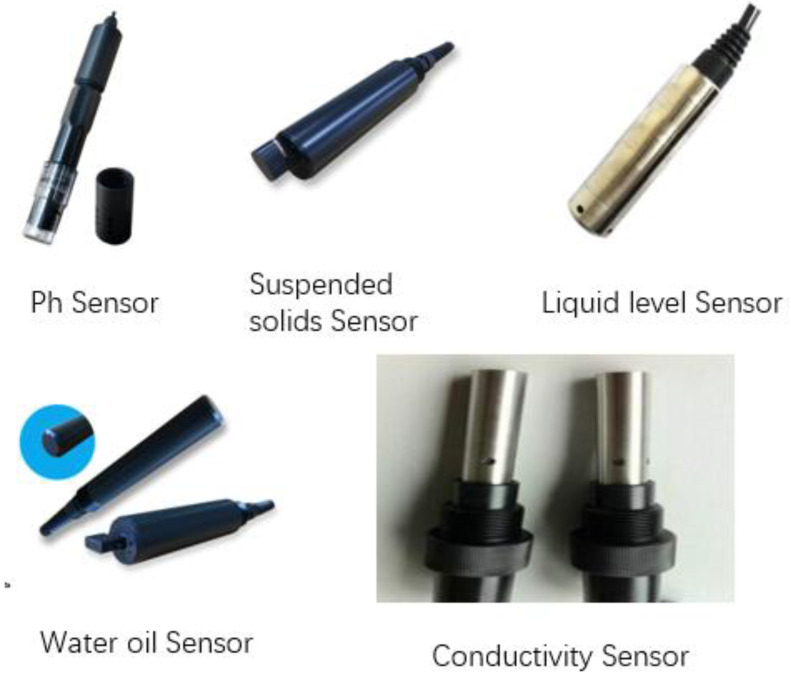
Water quality sensors.

(1) Mine water reuse environment sensing. Based on the perception of big data multi-data fusion, big data causality, and data mining and other advanced analysis technologies, this sensor continuously receives information related to water quality and water quantity, the status of open and closed valves, and the water demand of the mine area during the mine water treatment process. The sensor detects the underground–surface mine water treatment environment and the system operation status. The specific sensor is shown in Figure 19.
(2) Sensor data fusion analysis. Advanced analysis techniques, such as multiple data fusion [35,36], big data causality, and data mining, are applied to the sensed data to scientifically analyze various heterogeneous datasets based on their attributes and categories, providing information that can be utilized as a basis for intelligent and accurate judgment.
(3) Construction of mine water dispatch and reuse model. This is a mine water reuse model in the control system. Based on the fusion analysis of sensor data, this mathematical model can reflect actual information relating to the quantity and quality of mine water. In addition, the state of underground–surface water use is determined to intelligently allocate water resources in the mining area to achieve efficient and coordinated dispatch of underground water resources.

## 6. Conclusions

This paper presents and verifies a comprehensive design scheme of hierarchical allocation to improve the reuse rate of mine water. First, based on the mine water reuse system, a novel reuse scheduling strategy is proposed, a mathematical model of mine water optimal scheduling is established, and an improved particle swarm optimization algorithm is used to derive the model. The results show that the algorithm can optimize the water quality data collected by the sensor. In the heating and non-heating seasons, respectively, the reuse rate increased by 46.2% and 17.5%, the treatment time decreased by 147.59 and 117.51 h/month, and the reuse rate increased by 634.19 and 86 m^3^/h. In addition, the system has good adaptability to mine water reuse in different mining areas, ensuring excellent performance in water resource system deployment and water environment protection.

## Figures and Tables

**Table 1:** Corresponding table of mine water quality.

Variable Hierarchy	Water Point	Monthly Water Consumption/m^3^		Concentration/(mg/L)					Preset Variation Range (%)
		Heating Season	Non-Heating Season	SS	COD	Hardness	Oils	Turbidity	
**Underground—clean water tank**	Underground fire water	2880	2880	≤30	-	-	-	≤5	±10
	Grouting water	1840	576	≤30	-	-	-	≤5	±10
	Downhole dust removal water	144	288	≤30	-	-	-	-	±10
	Cooling water	720	720	≤30	≤60	≤450	≤1	≤5	±10
	Hydraulic support water	105.2	105.2	≤20	≤60	≤450	≤1	≤5	±10
**Pretreatment—intermediate tank**	Ground dust removal water	2304	2592	≤150	-	-	-	-	±20
	Fire water	96	2880	≤30	≤50	≤450		10	±10
**Secondary treatment—high tank**	Coal preparation water	1728	1296	≤400		≤500			±20
	Heat exchange station water	864	864	-		soft water			±25
	Cooling water	720	720	≤30	≤80	≤450	≤1	≤5	±20
	Greening water	288	288	-	≤50	≤450		≤10	±40
	Other domestic water	1440	1728	-	≤50	≤450		≤5	±40
**Advanced processing—reuse tank**	Boiler water	1728	288	≤30	≤60	≤450	≤1	≤5	±40
	Drinking water	57.6	57.6	Nothing visible	≤20	≤450	≤0.05	≤1	±40

**Table 2:** Treatment rate of mine water reuse points at all levels.

Mine Water Reuse Sites	Processing Speed (h/m^3^)	
	Heating Season	Non-Heating Season
Downhole—Clear water pool	4.48 × 10^−3^	4.48 × 10^−3^
Pretreatment—Intermediate pool	1.36 × 10^−3^	6.77 × 10^−4^
Secondary treatment—High-level tank	1.36 × 10^−3^	6.77 × 10^−4^
Deep processing—Multiplexed pools	1.36 × 10^−3^	6.77 × 10^−4^

**Table 3:** Test functions.

Test Function	Function Expression	Dimension	Search Scope	Meaning
Sphere	$f\left(x\right)=\sum_{i=1}^{D}x_{i}^{2}$	30	[–10, 10]	Global search capability
Rastrigin	$f\left(x\right)=\sum_{i=1}^{D}\left(x_{i}^{2}-10\cos\left(2\pi x_{i}\right)+10\right)$	30	[−10, 10]	Practicality
Rosenbrock	$f\left(x\right)=\sum_{i=1}^{D-1}\left[100{\left(x_{i+1}-x_{i}^{2}\right)}^{2}+{\left(x_{i}-1\right)}^{2}\right]$	30	[−10, 10]	Local search capability
Griewank	$f\left(x\right)=\frac{1}{4000}\sum_{i=1}^{D}x_{i}^{2}-\prod_{i=1}^{D}\cos\left(\frac{x_{i}}{\sqrt{i}}\right)+1$	30	[−10, 10]	Beyond local restrictions

Unified selection of experiments $\omega_{max}\;$= 0.95,$\;\omega_{min}$ = 0.4, *C*_1_ = *C*_2_ = 2.

**Table 4:** Test results.

Function	Pattern	LDIW-PSO	EDIW-PSO	ADIW-PSO
Sphere	Optimal value	6.665 × 10^−3^	9.88 × 10^−2^	4.336 × 10^−12^
	Average value	3.048 × 10^−1^	8.27 × 10^−1^	5.211 × 10^−9^
Rastrigin	Optimal value	3.095 × 10^−1^	2.668 × 10^−1^	5.819 × 10^+0^
	Average value	6.720 × 10^−1^	5.466 × 10^−1^	2.608 × 10^−1^
Rosenbrock	Optimal value	1.918 × 10^+0^	3.342 × 10^+0^	1.897 × 10^+0^
	Average value	7.933 × 10^+0^	7.745 × 10^+0^	6.934 × 10^+0^
Griewank	Optimal value	2.252 × 10^−7^	9.299 × 10^−5^	1.623 × 10^−5^
	Average value	4.798 × 10^−4^	4.064 × 10^−4^	2.797 × 10^−4^

**Table 5:** Water quantity of water consumption points in the mining area.

Mine Water Reuse Grade	Mine Water Reuse Point	Heating Season	Non-Heating Season
Underground treatment clear water tank	Underground fire fighting	65,735.22073	85,125.85209
	Grouting water	39,833.105	17,084.23175
	Underground watering and dust removal	3916.148483	8607.025781
	Cooling water	16,007.81491	20,629.249
	Hydraulic support	2321.444076	3084.392126
Pretreatment middle tank	Ground dust removal	85,951.97709	77,688.64651
	Fire water	106,881.3539	77,298.12662
Secondary treatment—high level tank	Coal treatment water	48,634.14471	36,839.152
	Heat exchange station water	14,866.30572	21,469.65259
	Cooling water	19,027.62374	20,063.64865
	Greening water	11,815.05183	8218.430315
	Other water use	34,813.89783	47,655.63901
Deep treatment reuse tank	Boiler water	80,165.00478	8252.076366
	Life Drinking	2076.096765	1727.431289

**Table 6:** Reuse amount of mine water at reuse points.

Water Reuse Points at All Levels of the Mine (N)	Recycling Volume in Traditional Mode m^3^/Month		Optimized Scheduling System Reuse m^3^/Month	
	Heating Season	Non-Heating Season	Heating Season	Non-Heating Season
Clear water tank (1)	160,770	160,770	12,7813.73	134,530.75
Middle tank (2)	0	0	192,833.33	154,986.77
High level tank (3)	0	0	129,157.02	134,246.52
Reuse tank (4)	51,840	58,875	82,241.10	9979.51

**Table 7:** Analysis of data before and after system optimization in the heating season.

Mode	Mine Water Reuse Rate (Month)	Mine Water Reuse Time (Month)	Reuse Speed (m^3^/h)
Traditional Scheduling	30.75%	720 h	295.29
ADIW-PSO	76.95%	572.41 h	929.48
LDIW-PSO	76.95%	620.18 h	857.89
EWIW-PSO	76.95%	615.21 h	864.82

**Table 8:** Data analysis before and after system optimization in the non-heating season.

Mode	Mine Water Reuse Rate (Month)	Mine Water Reuse Time (Month)	Reuse Speed (m^3^/h)
Traditional Scheduling	17.95%	720 h	305.06
ADIW-PSO	35.45%	602.49 h	719.92
LDIW-PSO	35.45%	666.49 h	650.79
EDIW-PSO	35.45%	658.82 h	658.38

## Data Availability

Not applicable.
